# Establishing Sustainable Cell Lines of a Coral, *Acropora tenuis*

**DOI:** 10.1007/s10126-021-10031-w

**Published:** 2021-04-26

**Authors:** Kaz Kawamura, Koki Nishitsuji, Eiichi Shoguchi, Shigeki Fujiwara, Noriyuki Satoh

**Affiliations:** 1 Department of Applied Science, Kochi University, Kochi, 780-8520 Japan; 2 Marine Genomics Unit, Okinawa Institute of Science and Technology Graduate University, Onna, Okinawa 904-0495 Japan

**Keywords:** Coral, Planula larvae, Cell dissociation, Cell lines, Single-cell RNA-seq

## Abstract

Planula larvae of the scleractinian coral, *Acropora tenuis*, consist of elongated ectodermal cells and developing inner endodermal cells. To establish in vitro cell lines for future studies of cellular and developmental potential of coral cells, larvae were successfully dissociated into single cells by treating them with a tissue dissociation solution consisting of trypsin, EDTA, and collagenase. Brown-colored cells, translucent cells, and pale blue cells were the major components of dissociated larvae. Brown-colored cells began to proliferate transiently in the culture medium that was devised for the coral, while translucent cells and pale blue cells decreased in number about 1 week after cell dissociation. In addition, when a modular protease, plasmin, was added to the cell culture medium, brown-colored cells extended pseudopodia and assumed amorphous shapes. They then continued to proliferate in clumps for more than 6 months with a doubling time of approximately 4–5 days. From 3 weeks of cell culture onward, brown-colored cells often aggregated and exhibited morphogenesis-like behavior to form flat sheets, and blastula-like clusters or gastrula-like spheres. Single cells or cell-clusters of the cell lines were analyzed by RNA-seq. This analysis showed that genes expressed in these cells in vitro were *A. tenuis* genes. Furthermore, each cell line expressed a specific set of genes, suggesting that their properties include gastroderm, secretory cells, undifferentiated cells, neuronal cells, and epidermis. All cell properties were maintained stably throughout successive cell cultures. These results confirm the successful establishment of a coral in vitro cell line.

**Supplementary Information:**

The online version contains supplementary material available at 10.1007/s10126-021-10031-w.

## Introduction

Animal in vitro cell lines have provided experimental systems for studies of developmental biology, regeneration, trans-differentiation biology, medical biology, and pharmaceutical biology. Such systems provide unambiguous answers to biological questions at the single-cell level. Although in vitro systems derived from mammals have been successfully established, creating cell lines from marine invertebrates is still challenging (Rinkevich 2011). Cells dissociated from tissues of marine invertebrates are not able to survive in seawater or in conventional culture medium for mammalian cells (Domart-Coulon and Ostrander 2016). In a well-known example from our studies of the tunicate, *Polyandrocarpa misakiensis*, we tried various culture media to establish cell lines (Kawamura and Fujiwara 1995). To our knowledge, there are no reports of successful establishment of a sustainable in vitro cell line of a stony reef-building coral (Domart-Coulon and Ostrander 2016), although primary cultures of cells or cell aggregates have been created for several anthozoans (Frank et al. 1994; Kopecky et al. 1999; Domart-Coulon et al. 2004; Khalesi 2008; Reyes-Bermudez 2009; Auzoux-Bordenave and Domart-Coulon 2010; Mass et al. 2012).

We undertook this project for the following reasons. First, coral reefs create the most diverse marine ecosystems, harboring about one fourth of all marine species (Wilkinson 2008), but coral reefs have been badly damaged by bleaching caused by increasing surface seawater temperatures, acidification, and pollution of oceans (Hoegh-Guldberg et al. 2007; Hughes et al. 2017). The keystone species of coral reefs are scleractinian corals, which produce rocky reefs by depositing calcium carbonate skeletons. Corals form obligatory endosymbioses with photosynthetic dinoflagellates of the family Symbiodiniaceae. Corals incorporate these dinoflagellates into their endodermal cells, but not ectoderm cells. In these endosymbioses, corals provide shelter for their algal symbionts, which supply the majority of their photosynthetic products to the host corals (Yellowlees et al. 2008). Therefore, understanding cellular and molecular mechanisms of coral-symbiont endosymbioses is essential for future projects regarding coral reef sustainability and restoration. Although many studies have attempted to explore cellular and molecular mechanisms of coral-symbiont endosymbiosis (Dove 2004; Shinzato et al. 2014), many questions remain, especially regarding recognition mechanisms involved in the initial contact of animal and algal cells, allowing dinoflagellate endocytosis, and maintenance of endosymbiosis. If we are able to establish coral cell lines that support endosymbiosis, we may be able to discover enabling mechanisms at the single-cell level.

Second, corals are cnidarians, which include *Hydra* and *Nematostella*. Cnidarians are the simplest Eumetazoans (animals with a tissue grade of organization), and their bodies consist of two germ layers, ectoderm and endoderm, with differentiated epithelial cells, neurons, stem cells, a complex extra-cellular matrix, muscle fibers, and a fixed axis of symmetry. Therefore, cnidarians provide an experimental system to examine molecular and cellular mechanisms involved in determining fates of primitive cells, from which different cell types develop. Being able to do this in vitro and/or at the single-cell level will greatly advance our knowledge of basic developmental biology.

**Fig. 1:**
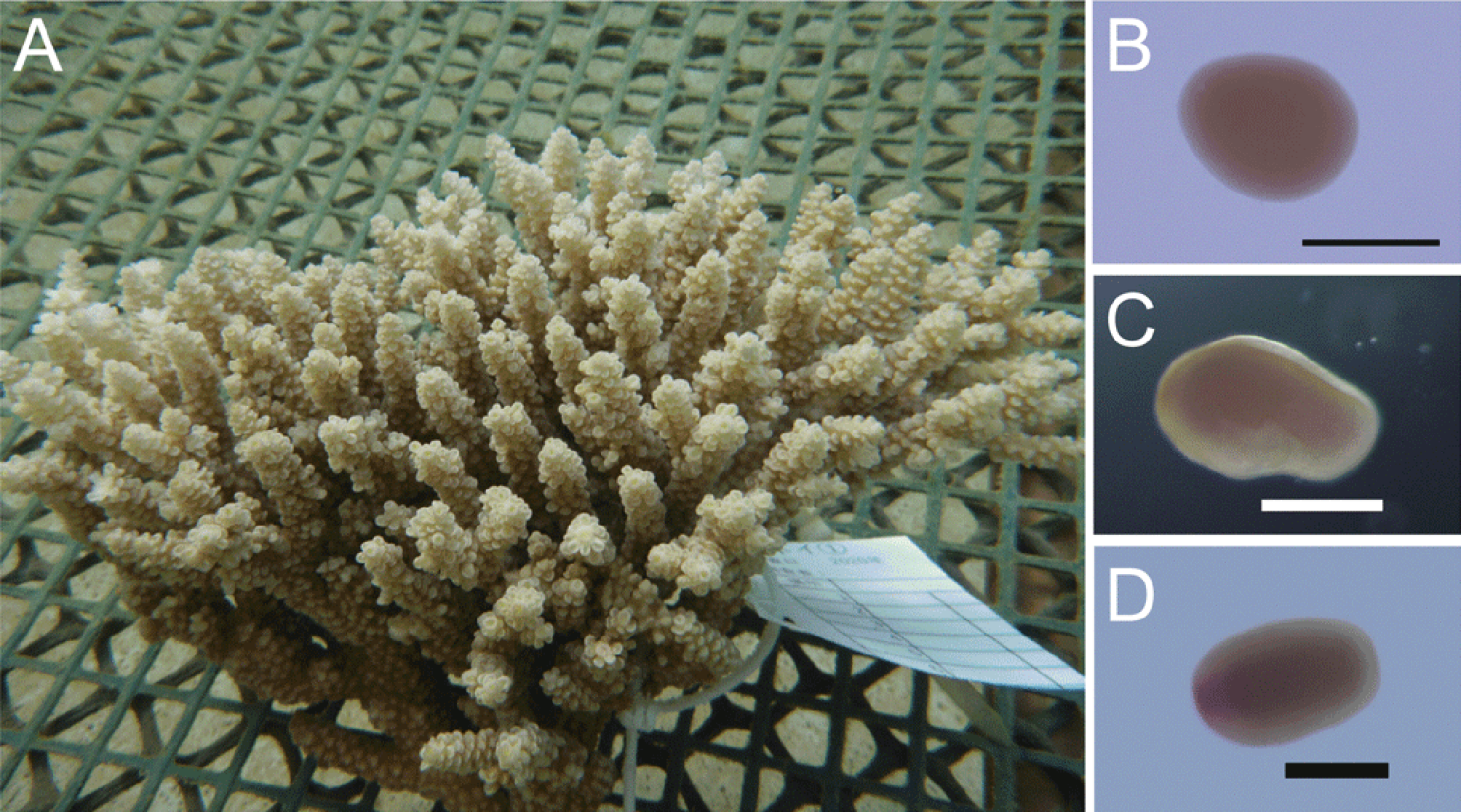
The coral, *Acropora tenuis*. **a** An adult colony. **b** A 3.5-day-old embryo. **c** A 5.5-day-old planula. **d** A 36-day-old planula. The left portion of the larva looks pinkish and corresponds to the oral side. Scale bars, 250 μm

We selected planula larvae of the coral, *Acropora tenuis* (Fig. 1), as the focus of this study, since *A. tenuis* is one of the most abundant species in coral reefs. Moreover, its genome has been decoded (Shinzato et al. 2020), and we reasoned that larval cells might have more cell growth potential in vitro than adult cells.

## Materials and Methods

### Biological Materials

With permission from Okinawa Prefecture, colonies of *Acropora tenuis* (class Anthozoa; sub-class Hexacorallia, order Scleractinia, family Acroporidae) with mature gonads were collected at Sekisei Lagoon near Ishigaki Island in late March 2019 and along the Onna Coast of Okinawa Island in mid-May 2020 (Fig. 1a). Collected colonies were stored in aquaria at the Research Center for Subtropical Fisheries, Seikai National Fisheries Research Institute, Ishigaki, and in aquaria at the Onna Fishery Port, until spawning started. To adjust timing of fertilization in different batches, one or two hours after signs of spawning appeared, individual colonies were transferred to separate small aquaria, from which egg and sperm bundles were collected. After bundles were broken and gametes were released, eggs and sperm from different colonies were mixed to obtain successful fertilization, followed by synchronous development of embryos and larvae.

Embryos and larvae were cultured in filtered seawater (FSW) in the laboratory at room temperature (~ 25 °C). FSW was made with a Sartolab RF 1000 Filter System (Sartorius) with a 0.22-µm PES filter. Larvae were maintained in Cambro Round Food Containers filled with 2L of FSW, which was exchanged every day for the first week and then once every other day after 8 days post-fertilization. At the time of FSW exchange, we confirmed that there was no protist contamination of the culture medium.

Several batches of larvae were transferred to Kochi University and used for serial cell dissociation at 3 (Fig. 1b), 5 (Fig. 1c), 10, 12, 14, 16, 24, 36 (Fig. 1d), and 57 days after fertilization.

### Cell Dissociation

Trypsin/EDTA (T4049-100ML, Sigma) was dispensed in 15-mL conical tubes and preserved in the freezer. Collagenase type I (Co130-100MG, Sigma) was dissolved in sterile water at a concentration of 100 μg/mL. Immediately before use, a stock solution of collagenase was added to trypsin/EDTA at a final concentration of 1 μg/mL. Planula larvae (20–40 individuals) were collected from the surface of planula container with Pasteur pipettes and digested with more than 10 volumes of trypsin/EDTA/collagenase (TEC) solution for 1–4 h at 25–28 °C in a rocking incubator.

### Cell Culture Medium

The cell culture method was modified from the protocol developed for the marine invertebrate, *Polyandrocarpa misakiensis* (class Ascidiacea; family Styelidae) (Kawamura and Fujiwara 1995).

Briefly, the basic seawater medium consisted of natural seawater, one-fifth volume of H_2_O, 10 mM HEPES (final pH 6.8), and the antibiotics penicillin (100 U/mL), streptomycin (100 μg/mL), and amphotericin B (0.25 μg/mL). Immediately before use, the basic medium was mixed with Dulbecco’s modified Eagle medium (DMEM) containing 15% fetal bovine serum, penicillin (100 U/mL), and streptomycin (100 μg/mL) at a ratio of 9:1. In the secondary cell culture, plasmin (166-24231, FUJIFILM Wako Pure Chemical Corp., Osaka, Japan) was added to the growth medium at a final concentration of 2 μg/mL.

Dissociated cells were centrifuged and resuspended in the growth medium at a density of 2–5 × 10^7^ cells/mL. Aliquots (0.5 mL each) were dispensed to a 24-well multiplate and maintained at 20 °C by adding 0.2 mL of fresh growth medium to the old medium twice a week. Proliferating cells were replated in new multiplates every month.

### Cell Growth Kinetics

The calculation of doubling time was carried out using a modified tetrazolium method (Kawamura et al. 1999). Thiazolyl blue tetrazolium bromide (MTT) (M2128, Sigma) was dissolved at a concentration of 5 mg/mL in sterile phosphate-buffered saline (PBS). Aliquots of cell suspension (0.7 × 10^4^ cells/0.5 mL) were dispensed to all wells of a 24-well multiplate, and 50 μL of the MTT stock solution was added to four wells of the plate every 2 days for 12 days. Four hours after MTT administration, 0.5 mL of 10% sodium dodecyl sulfate (SDS) in 0.01 N HCl was added to wells containing MTT to solubilize developing formazan by overnight incubation. Absorbance at 600 nm and the reference at 690 nm were measured.

### Histology

Planula larvae were fixed in Zamboni’s fixative (2% paraformaldehyde, 15% saturated picric acid, 0.15 M phosphate buffer (PB) (pH 7.3)) for 1 h in an ice bath, and then serially dehydrated and infiltrated with a plastic resin, Technovit 8100 (64709012, Heraeus Kulzer, Wehrheim, Germany) for more than 10 h at 4 °C. After hardening, samples were sectioned into 2-µm slices with glass knives. For general histology, sections were stained with 0.5% toluidine blue in 0.1 M PB (pH 7.4) for 5 min.

### Antibodies and Immunohistochemistry

To confirm that dissociated and cultured cells are derived from *Acropora tenuis* larvae, we made antibodies against two *A. tenuis* proteins, Snail and Fat1. Snail is a zinc finger transcriptional repressor of the Snail gene superfamily that participates in development and survival of cells (Nieto 2002). The synthetic oligopeptide, RERNASTSDVSQRK, corresponding to amino acids 135–148 of the Snail protein of *A. tenuis* (AtSnail), was conjugated to the carrier protein, keyhole limpet hemocyanin (KLH). Rabbit anti-AtSnail antibody was raised (Eurofins Genomics, Tokyo, Japan) and diluted 400-fold with PBS immediately before use. If necessary, the antibody was adsorbed to KLH.

Anti-AtFat1 rabbit antibody was also prepared from the synthetic oligopeptide, KKKREQRAQEEAAK, corresponding to amino acids 4259–4272 of AtFat1 protein. Secondary antibodies labeled with horseradish peroxidase (PI-1000) (Vector Laboratory, Burlingame, CA, USA) were diluted 200-fold with PBS.

To confirm the antibody specificity, we carried out Western blot analysis of total proteins of *Acropora tenuis.* Total proteins were isolated from planula larvae (8 days after fertilization) and from culture cells according to a standard procedure. Isolated proteins were analyzed with SDS-PAGE gel electrophoresis and then transferred to membranes. Membranes were incubated in a mixture of 0.25% blocking reagent (Roche, Mannheim, Germany) and 5% skim milk in PBS for 1 h. Then, they were incubated with primary antibody diluted 1000-fold for 1 h and with secondary antibody diluted 200-fold for 0.5 h.

For enzyme immunohistochemistry, sections were incubated in a mixture of 0.25% blocking reagent (Roche, Mannheim, Germany) and 5% skim milk in PBS for 30 min. Then, they were incubated with the primary antibody for 1 h and with the secondary antibody for 0.5 h. Sections were stained with TrueBlue (KPL).

### RNA-seq Analysis

Individual cultured cells or cell clusters were collected using handmade glass micropipettes under a binocular microscope (Leica, model: PN: MDG33/10450123) and dropped into 10-µL PCR tubes filled with TaKaRa SMART-seq HT (Takara Bio Inc., Shiga, Japan). The TaKaRa SMART-Seq HT cDNA-synthesis kit allows us to prepare libraries from as little as 10 pg of total RNA. This kit also contains a poly(A) selection step using an oligo(dT) primer, enabling researchers to remove contaminating rRNAs. Then RNA-seq libraries were created using Nextera XT kits and IDT for Illumina Nextera UD indexes Set A (96 indexes, 96 libraries) (Illumina Inc., CA, USA). They were sequenced using NovaSeq 6000 SP reagent.

Genome data of *A. tenuis* was downloaded from https://marinegenomics.oist.jp/acropora_tenuis/viewer/info?project_id=97. Adapter sequences of RNA-seq reads were trimmed using trimmomatic software (version 0.30) (Bolger et al. 2014). Trimmed RNA-seq reads were mapped to *A. tenuis* mRNA data using Salmon software (version 0.8.2) (Patro et al. 2017). Specific genes of each cell were identified using marker genes described by Sebe-Pedros et al. (2017). Transcriptome abundance was estimated as transcripts per million (TPM). Genes mapped by RNA-seq reads were annotated using InterProScan (5.46-81.0) software.

### GO Annotation

GO annotation analysis of genes expressed in culture cells was carried out using InterProScan (EMBL-EBI).

## Results

### Cell Components and Features of Planula Larvae

At room temperature (about 25 °C), fertilized eggs of *Acropora tenuis* cleave and develop into prawn chip-shaped blastulae (15 h after fertilization), donut-like gastrulae (1 day after fertilization), pear-shaped embryos (3.5 days after fertilization; Fig. 1b), and planula larvae (5.5 days after fertilization; Fig. 1c). We maintained planula larvae more than 1 month in the laboratory (Fig. 1d).

**Fig. 2:**
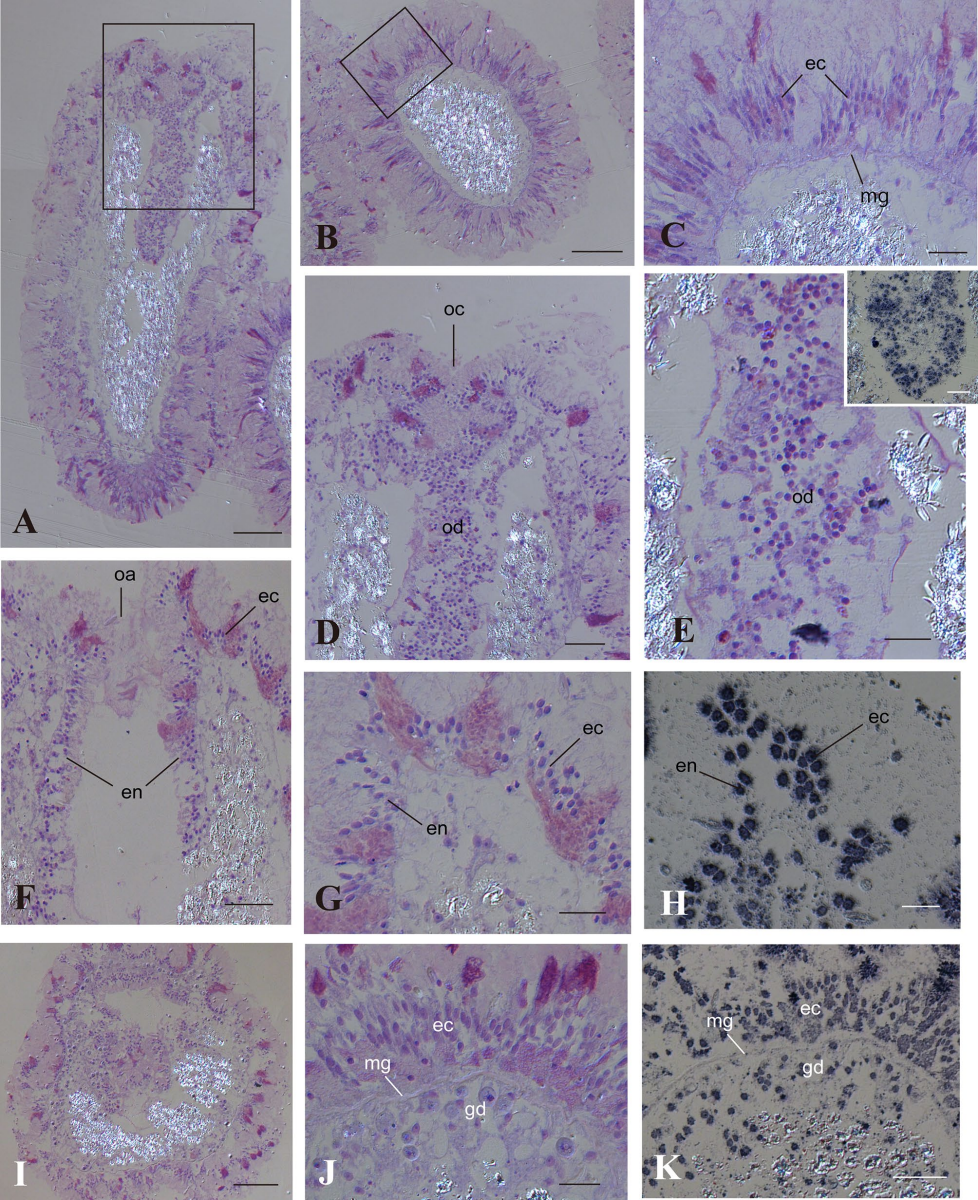
Anatomy of *Acropora tenuis* planula larvae. **a**–**h** A 24-day-old planula larva. **a** Longitudinal section with the oral concavity located at the top. Bar, 100 μm. **b** Transverse section. Bar, 100 μm. **c** Higher magnification of a rectangle region in **b**. Note that endoderm was not observed beneath the mesoglea. Bar, 20 μm. **d** Higher magnification of the oral concavity shown by a rectangle in **a**. Bar, 40 μm. **e** Oral diverticulum. Note that constituent cells are loosely associated. Bar, 20 μm. (Inset) Anti-AtSnail immunostaining of the oral diverticulum. Bar, 20 μm. **f** Oral aperture just before opening in a 24-day-old planula larva. Note that the diverticulum has a hollow space and that constituent cells access the ectoderm. Bar, 40 μm. **g** Oral ectoderm and diverticulum. Bar, 20 μm. **h** Anti-AtSnail immunostaining of oral ectoderm and diverticulum. Bar, 20 μm. **i**–**k** A 67-day-old planula larva. **i** Transverse section. Bar, 100 μm. **j** Higher magnification of planula body wall. Bar, 20 μm. **k** Anti-AtSnail immunostaining of planula body wall. Bar, 20 μm. ec ectoderm, en endoderm, gd gastroderm, mg mesoglea, oa oral aperture, oc oral concavity, od oral diverticulum

In order to learn more about basic cellular components and morphogenesis of *A. tenuis* planula larvae, histological sections were prepared from 24-day-old and 57-day-old planula larvae (Fig. 2). Twenty-four-day-old larvae are beginning to form the oral diverticulum or pharynx while 57-day-old larvae have completed pharynx formation. A transverse section of a 24-day-old larva shows that the ectoderm consists of especially elongated cells (Fig. 2b, c). Endoderm was not observed beneath the ectoderm at this stage (Fig. 2c). A longitudinally cut surface shows a diverticulum hanging from the oral concavity (Fig. 2a). The diverticulum is continuous with invaginated ectoderm (Fig. 2d, f, g). Cells in the diverticulum are loosely associated (Fig. 2e). Some 24-day-old planula larvae were beginning to open the oral aperture (Fig. 2f, g). The diverticulum has a cavity at the center (Fig. 2g) and constituent cells of the diverticulum have approached the wall of the ectoderm to form an endodermal cell layer (Fig. 2g). Histological sections of 67-day-old planula larvae show that ectoderm is already underlain by endoderm, the presumptive gastroderm of a forthcoming polyp (Fig. 2i, j).

### Cell Dissociation and the Primary Cell Culture

**Fig. 3:**
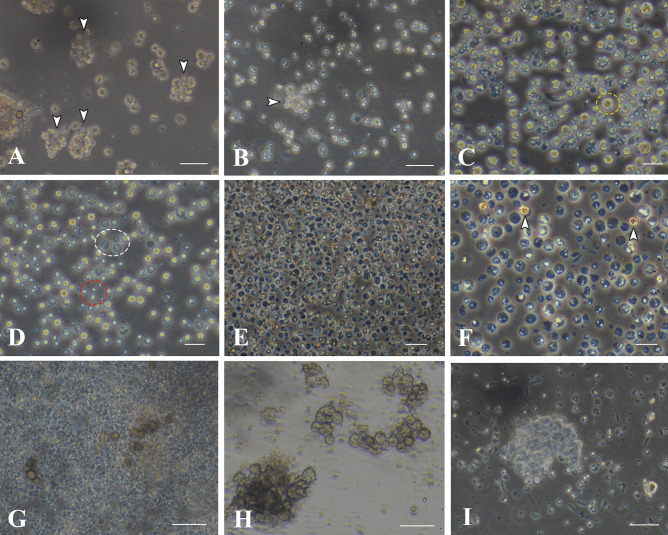
Primary cell culture in the absence of plasmin. Cells were dissociated from 5-day-old planula larvae except **e** and **f**, which were taken from 57-day-old larvae. **a** Cells treated with TEC for 1 h. Arrowheads show tissue debris. Bar, 40 μm. **b** Cells treated with TEC for 2 h. Arrowheads show debris. Bar, 40 μm. **c** Cells treated with TEC for 4 h. A dashed yellow circle shows a brilliant brown cell. Bar, 20 μm. **d** Cells treated with TEC for 4 h, and 2 days after dissociation. A dashed white circle shows translucent cells. A dashed red circle shows small, pale, blue cells. Bar, 20 μm. **e** Cells after 1 day cultivation. Bar, 40 μm. **f** Higher magnification of **e**. Note that brown cells (white arrowheads) are scarcely seen. Bar, 20 μm. **g** Cells after 7 days cultivation. Bar, 40 μm. **h** Cells after 12-day cultivation. Bar, 40 μm. **i** Cells 32 days after dissociation. Bar, 40 μm

Forty to 50 larvae were collected 3, 5, 10, 12, 14, 16, and 24 days after fertilization. They were treated with a mixture of trypsin, EDTA, and collagenase (TEC). TEC treatment for 1–2 h at 25–28 °C did not complete cell dissociation (Fig. 3a, b, arrowheads), but after 3–4 h, only single cells without debris were found in the culture dish (Fig. 3c). Irrespective of larval ages, TEC efficiently dissociated cells, whereas TE was less effective, and collagenase alone had no apparent effect on cell dissociation (data not shown). The dissociated cell population contained several types of cells (Fig. 3c, d), including brilliant, brown-colored cells approximately 10 μm in diameter (Fig. 3c), translucent cells 8–10 μm in diameter (Fig. 3d), and small, pale blue cells (2–4 μm in diameter) that may be cell fragments rather than intact cells (Fig. 3d). Elongated cells characteristic of live ectoderm were scarcely found in the culture dish (Fig. 3a–d). We performed cell dissociation on 57-day-old and 61-day-old larvae as well. Cell populations dissociated from these stages contained a very few brown cells (Fig. 3e, f), as will be discussed later.

Dissociated cell suspension (0.5 mL) was dispensed in each well of a 24-well plate. Small cell aggregates appeared 4 to 7 days later (Fig. 3g). Cell aggregates increased in number and size until 2 weeks of incubation (Fig. 3h) and then stopped growing without any signs of cell death (Fig. 3i).

### Effect of Plasmin on Cell Growth

**Fig. 4:**
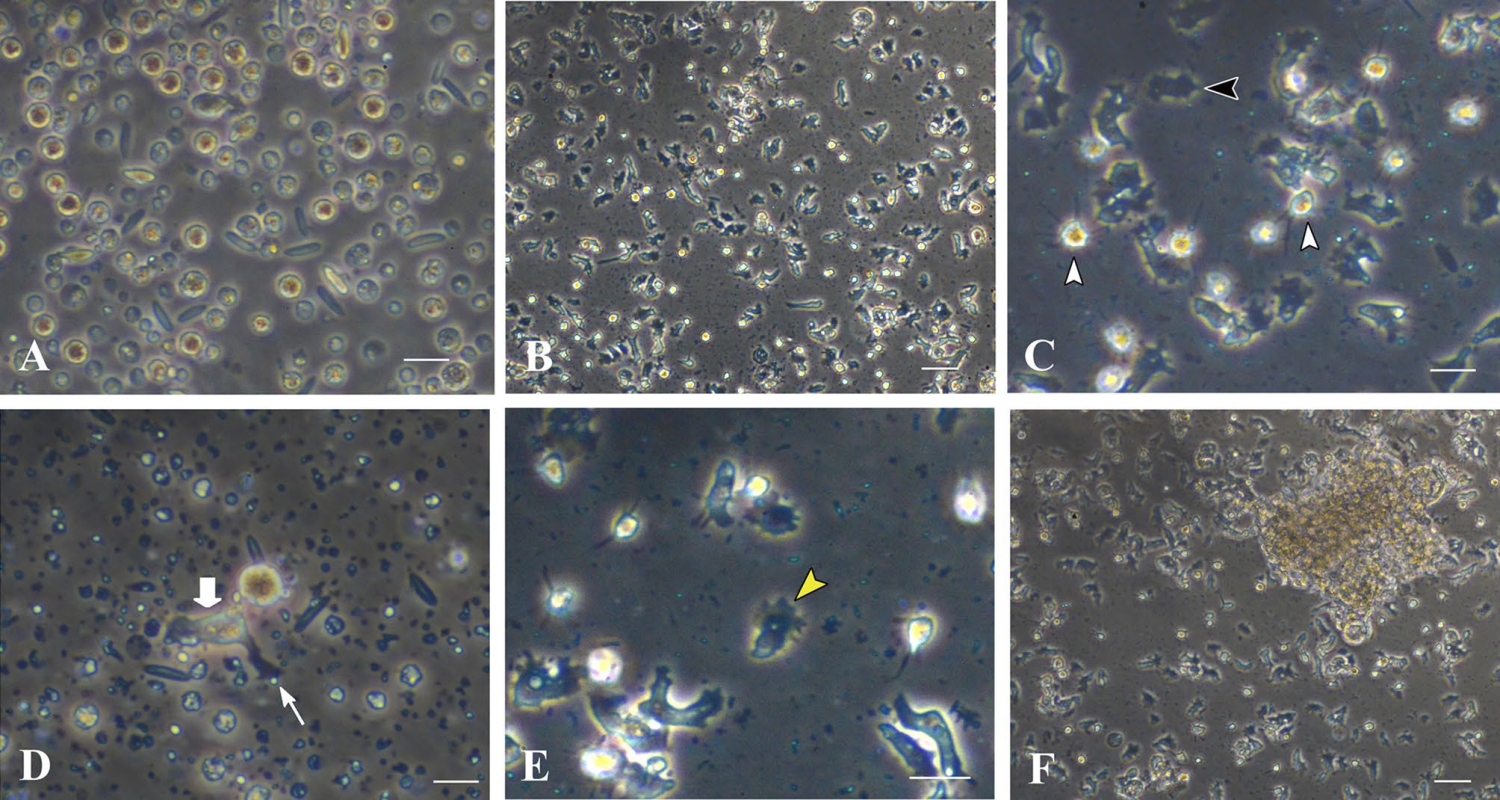
Primary cell culture of 24-day-old planula larvae in the presence of plasmin. **a** Cells treated with TEC for 4 h. Bar, 20 μm. **b** Cells after 13-day cultivation. Bar, 50 μm. **c** Higher magnification of brown cells (white arrowheads) and dark amorphous cells (black arrowhead). Brown cells bear well-developed filopodia (white arrowheads). Bar, 20 μm. **d** Cells of intermediate-like stage between brown cells (thick arrow) and dark cells, having elongated lamellipodia (thin arrow), 17 days after cell dissociation. Bar, 20 μm. **e** Dark amorphous cells with lamellipodia (yellow arrowhead), after 17-day cultivation. Bar, 20 μm. **f** Reaggregation of brown cells, 20 days after cell dissociation. Bar, 50 μm

Modular proteases were applied to the primary cell culture in the basal growth medium immediately after cell dissociation, because modular proteases such as tunicate retinoic acid-inducible modular protease (TRAMP) (Ohashi et al. 1999) and enterokinase (Kawamura et al. 1999) sometimes exhibit cell proliferation activity in invertebrate cell cultures. Enterokinase had no apparent effects on planula cell growth. In contrast, plasmin (2 μg/mL) was effective in maintaining dissociated cells in culture for more than 2 weeks (Fig. 4a, b), during which a large number of a new type of cell appeared in the culture dish (Fig. 4a, b). The new type of cell looked dark and had an amorphous shape (Fig. 4c, yellow arrowhead). After 2 to 3 weeks of culture, brown cells extended filopodia (Fig. 4c, white arrowheads) and dark cells developed lamellipodia (Fig. 4e, black arrowhead). The intermediate type of cell having brown-colored cell body (Fig. 4d, thick arrow) and elongated lamellipodium (Fig. 4d, thin arrow) was often observed, suggesting that brown cells differentiate in vitro into dark cells. Brown cells formed loose cell aggregates similar to the oral diverticulum of planula larvae (Fig. 4f). The aggregate is easily dissociated into single cells by pipetting.

**Fig. 5:**
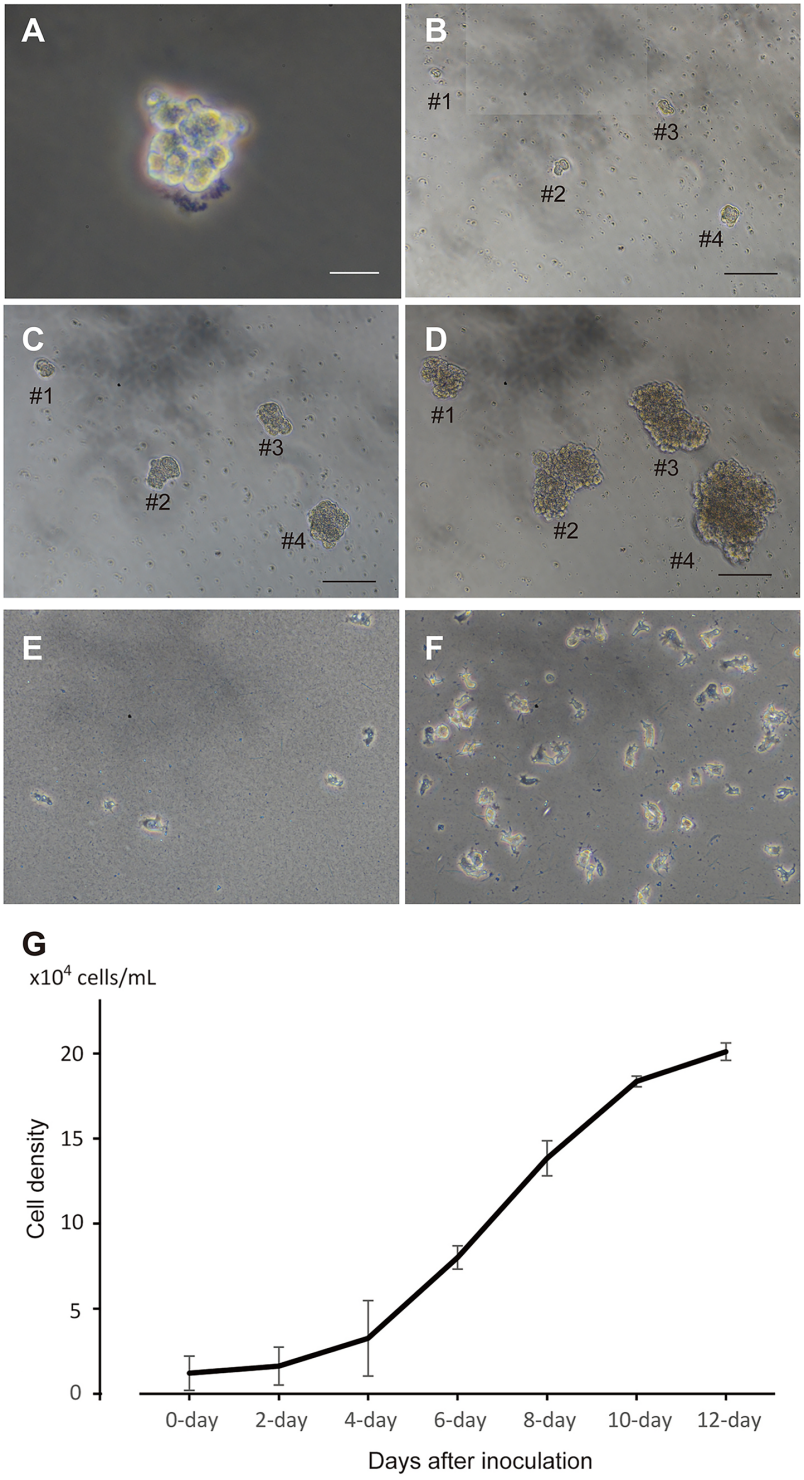
Cell growth in the secondary cell culture. **a** A small cell clump immediately after inoculation. Bar, 20 μm. **b**–**d** Serial observation of cell clumps. Bar, 100 μm. **b** Five days after inoculation. **c** Seven days after inoculation. **d** Ten days after inoculation. **e**–**g** Growth curve of cells and appearances of growing cells after 1 day **e** and 12 days **f** of cultivation. **g** Cell growth curve analyzed by the MTT method. **f** Twelve days after inoculation

For subsequent cell culture, aliquots of polyclonal cell population were harvested from the primary 24-well culture plate, diluted fivefold with growth medium containing plasmin, and dispensed into 96-well plates (100 μL/well). Cells in clumps (Fig. 5a) proliferated with a doubling time of 2–3 days (Fig. 5b–d). For quantitative analysis of cell growth, the scattering type of cell was inoculated in a multiplate at a cell density of 1.5 × 10^4^ cells/mL (Fig. 5e). Following an initial stagnation of cell growth, cells approximately doubled their original cell density every 2 days from 4 to 8 days of cell culture (Fig. 5f, g). Then, cell growth slowed, and they doubled in 4 days from 8 to 12 days of cell culture (Fig. 5g). In all, cells proliferated eightfold after 12 days of culture (Fig. 5f, g).

### Making Cell Lines

**Fig. 6:**
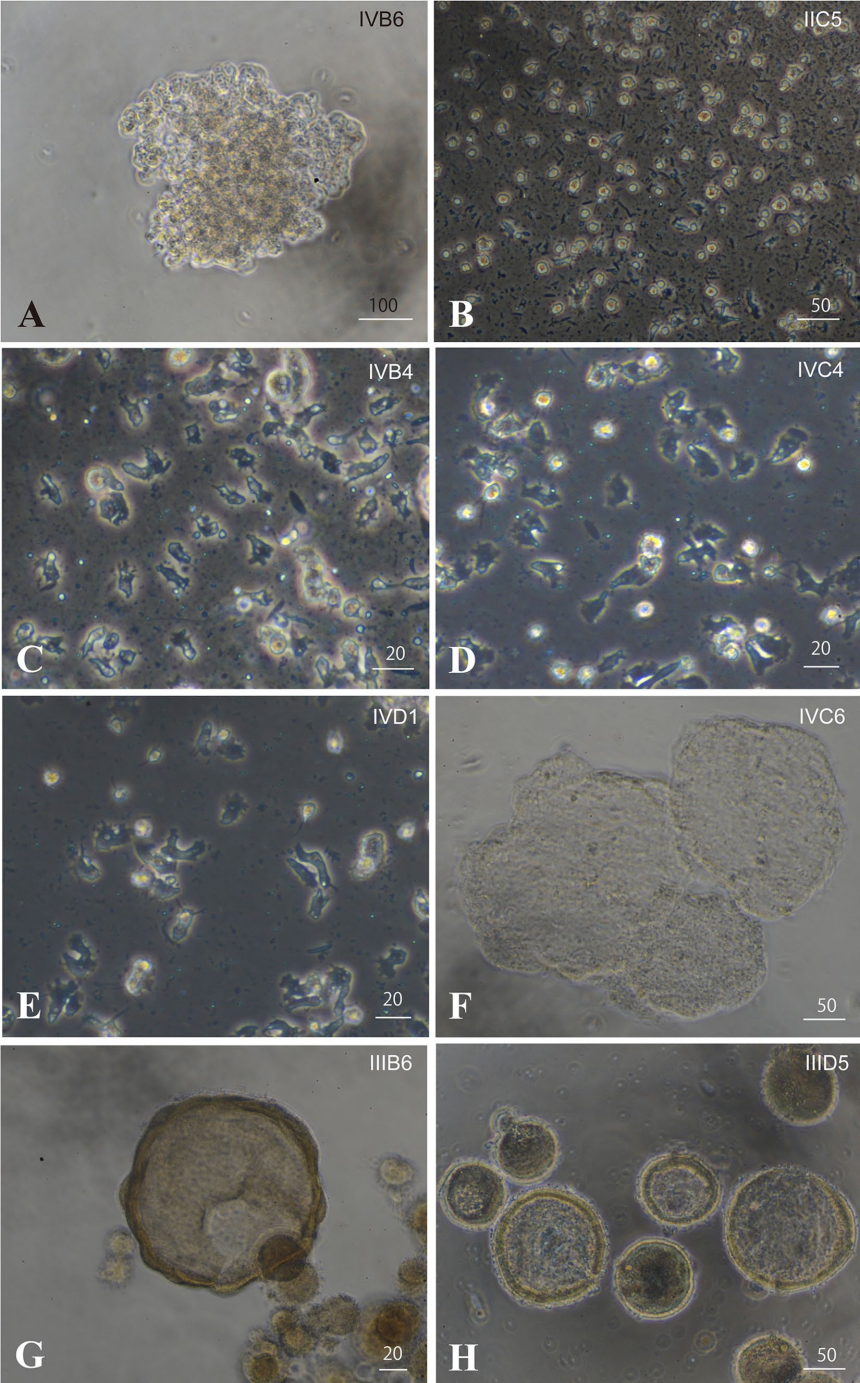
Eight representative in vitro cell lines made by the present study. **a**, **b** Brilliant brown cell lines. **a** Line IVB6 forming a cluster, whereas IIC5 cells (**b**) were stable as single cells. **c**–**e** Flattened, dark, amorphous cell lines, **c** IVB4, **d** IVC4, and **e** IVD1. **f** IVC6 tends to form cell sheets. **h** IIID5 forms blastula-like cell clusters. **g** IIIB6 develops into gastrula-like cell clusters. Bar in **a**, 100 μm. Bars in **b**, **f**, and **g**, 50 μm. Bars in **c**–**e** and **h**, 20 μm

From 3 weeks onward, some cell aggregates formed cell sheets (Fig. 6a, f). Other cell aggregates formed spheres (Fig. 6g) detached from the substratum. They resembled blastulas (Fig. 6g), and in the most extreme cases, gastrula-like aggregates appeared (Fig. 6h). Blastula-like and gastrula-like aggregates were returned to seawater, but they did not swim and never developed into planula larvae.

Therefore, so far, we have attempted to make several *A. tenuis* cell lines with different features. Eight representative lines and their features are listed in Table 1 and Fig. 6. Lines IVB6 and IIC5 are brilliant brown cells, which exist as single cells (Fig. 6b) or clusters (Fig. 6a). Lines IVB4 (Fig. 6c), IVC4 (Fig. 6d), and IVD1 (Fig. 6e) are dark, amorphous, flattened cells. IVC6 (Fig. 6f) has a propensity to form cell sheets. As mentioned above, IIID5 forms blastula-like cell clusters (Fig. 6h) and IIIB6 develops into gastrula-like cell clusters (Fig. 6g). These cell lines have been maintained for more than 8 months by replating them more than eight times (Table 1). In addition, the cells are cryo-preservable in liquid nitrogen. The cell lines are distributable to academic researchers upon request to Dr. Kaz Kawamura at Kochi University.

**Table 1:** Cell lines and morphological features of cells

Cell lines	Cell features	Date when cell culture begins	No. of times of replating*	Resource availability**
IVB6 (Fig. 6a)	Brilliant brown Aggregate Immovable	July 1, 2020	6	O
IIC5 (Fig. 6b)	Brilliant brown Single Filopodia Movable	June 21, 2020	8	O
IVB4 (Fig. 6c)	Dark, flattened Amorphous Lamellipodia Movable	July 1, 2020	7	O
IVC4 (Fig. 6d)	Dark, flattened Amorphous Lamellipodia Movable	July 1, 2020	7	O
IVD1 (Fig. 6e)	Dark, flattened Amorphous Lamellipodia Movable	July 1, 2020	7	O
IVC6 (Fig. 6f)	Bright, vacuoles Immovable, forms cell sheet	July 1, 2020	4	O
IIID5 (Fig. 6h)	Bright, vacuoles Immovable, forms blastula-like sphere	June 23, 2020	4	O
IIIB6 (Fig. 6g)	Bright, vacuoles Immovable, forms gastrula-like sphere	June 23, 2020	2	X

*As of February 12, 2021

**Cell lines except for IIIB6 are cryo-preservable and are distributable to academic researchers. Please contact Dr. Kaza Kawamura at Kochi University: e-mail, kazuk@kochi-u.ac.jp

### Immunochemical Proof of *A. tenuis* Cell Lines

We were confident that the established cell-lines originated from *Acropora tenuis* planula larvae. However, there was still a possibility that these cells represented contamination from unknown sources. To examine this possibility, we made two antibodies, anti-AtSnail and anti-AtFat1 (see Methods) and carried out Western blotting and immunohistochemistry using these antibodies. We first carried out Western blotting analysis against total proteins isolated from planula larvae or cultured cells and confirmed the specificity of the antibodies to *A. tenuis*. The analysis using anti-AtSnail antibody resulted in a band of approximately 30 kDa protein (Supplementary Fig. S1a, lanes 1, 2, asterisk), corresponding to the expected MW of AtSnail (29,813 Da). AtFat1 is expected to be an extraordinarily large protein of approximately 400 kDa. It is impossible to analyze this large protein by means of our SDS-PAGE system. In the present study, the anti-AtFat1 antibody reacted with a protein of approximately 200 kDa and with several minor polypeptides extracted from planula larvae (Supplementary Fig. S1b, lane 1, asterisk). The antibody did not stain any specific bands extracted from cultured cells (Supplementary Fig. S1b, lane 2). The results of anti-AtSnail antibody indicate that the antibody is specific for *A. tenuis* proteins.

Then, we carried out immunocytochemistry of 24-day-old and 67-day-old planula larvae. Nuclei of both ectoderm and endoderm were stained with anti-AtSnail antibody (Fig. 2e, h, k), and anti-AtFat1 stained cell bodies (data not shown).

**Fig. 7:**
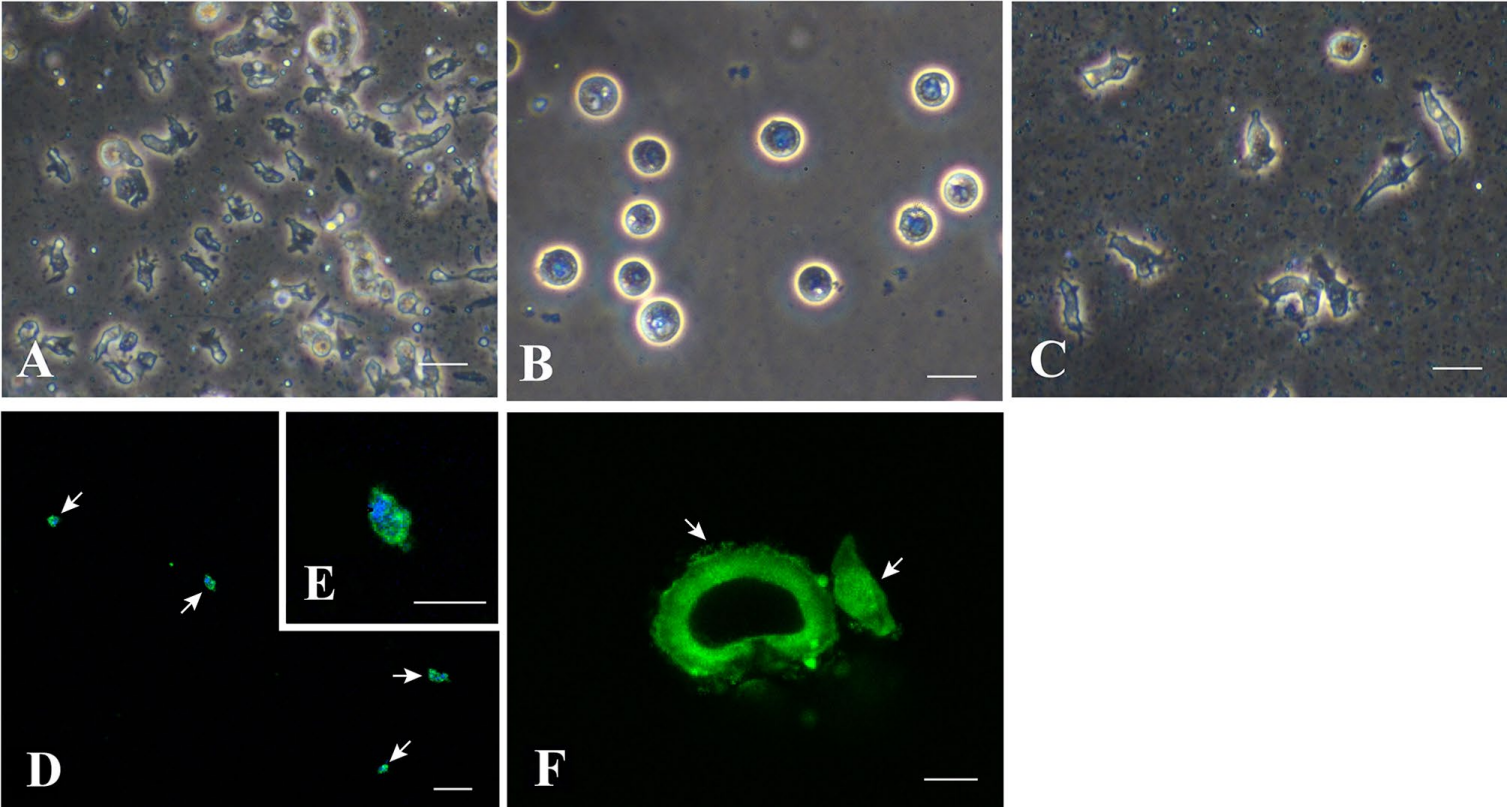
Immunostaining of in vitro cultured cells. **a** Cultured cells in growth medium. **b** Cells 5 min after being treated with TE. Note that all cells are detached from the substratum and have assumed a spherical form. **c** Cells 12 h after being returned to the growth medium. They extend pseudopodia at the bottom of the culture dish. **d**–**f** Fluorescent images of immunostaining cells. **d**, **e** Anti-AtSnail staining merged with a DAPI image. An arrowhead shows the nucleus. **f** Anti-AtFat1 immunostaining. Several gastrula-like cells clustered to form a hollow sphere. Bars in **a**–**c**, 20 μm. Bars in **d**, **e**, 5 μm. Bar in **f**, 40 μm

In vitro cultured cells (Fig. 7a) were treated with TE for 5 min. All cells were dissociated from the culture dish and assumed spherical shapes (Fig. 7b). When the spherical cells were inoculated again into cell growth medium containing plasmin, they adhered to the substratum within 12 h (Fig. 7c). This feature of cells was utilized to stick them to coverslips. Cultured cells on the coverslip were stained with anti-AtSnail antibody (Fig. 7d–f). Immunostaining was not restricted to the nucleus (Fig. 7e) but also occurred in the cytoplasm. Anti-AtFat1 antibody was also used to stain cell aggregates mentioned above (Fig. 7f). The fluorescent signal appeared to emit from the whole cell aggregate (Fig. 7f). These results indicate that cells are derived from *Acropora tenuis*.

### Molecular Characterization of Cell Lines

We carried out RNA-seq analyses of gene expression profiles of the eight cell lines listed in Table 1 and Fig. 6. Single-cell RNA-seq was performed on lines IVB6, IIC5, IVB4, IVC4, and IVD1 or a single-cell cluster of lines IVC6, IIID5, and IIIB6. For each cell line, the Illumina Novaseq platform yielded ~ 30 Gb of reads. Genome and transcriptome data of *A. tenuis* were downloaded from https://marinegenomics.oist.jp/acropora_tenuis/viewer/info?project_id=97. Single-cell RNA-seq reads were mapped to *A. tenuis* mRNA data using Salmon software (version 0.8.2) (Pertea et al. 2016). Data from this analysis are shown in Supplementary Table S1. Raw data of RNA-seq were deposited in GenBank and are accessible under BioProject ID, PSUB014043.

As a result, 902 mRNAs with corresponding gene models were identified, 676 of which were annotated with sequence similarities to those of other metazoans, while the remaining 226 resulted in no annotation. This confirmed the results of immunocytochemistry that the in vitro cell lines belong to *Acropora tenuis* and did not result from contamination.

Of 676 genes, 36 were expressed in all eight cell lines and 640 genes were expressed preferentially and/or specifically in a certain line. Further characterization showed that 54 genes were specifically expressed in IVB6, 71 gene in IIC5, 61 in IVB4, 120 in IVC4, 53 in IVD1, 146 in IVC6, 38 in IIID5, and 31 to IIIB6 (Supplementary Table 2). This indicates that these cell lines are not identical as far as can be judged from gene expression profiles.

**Table 2:** Expression of marker genes in eight cell lines

Tissue	Marker_genes	GeneID	IVB6	IIC5	IVB4	IVC4	IVD1	IVC6	IIID5	IIIB6	Annotation
Gastroderm	Collagen	aten_s0099.g45.t1	0	0	0	0	0	4.11	0	0	COMM domain-containing protein 8
Gastroderm	Collagen	aten_s0293.g4.t1	0	0	0	0	0	0.32	0	0	Peroxidasin homolog
Gastroderm	Collagen	aten_s0015.g70.t1	1.37	0	0	0	0	0	0	0	Calcium/calmodulin-dependent protein kinase type II delta 1 chain
Gastroderm	Collagen	aten_s0117.g39.t1	0.33	0	0	0	0	0	0	0	RNA polymerase-associated protein LEO1
Gastroderm	Collagen	aten_s0135.g41.t1	0.24	0	0	0	0	0	0	0	Neurogenic locus Notch protein
Gastroderm	Collagen	aten_s0078.g21.t1	0	0	0	0.07	0	0	0	0	Collagen alpha-4(VI) chain
Gastroderm	Collagen	aten_s0079.g120.t1	0	0	2.35	0	8.11	0	0	0	Hippocampus abundant transcript 1 protein
Gastroderm	Collagen	aten_s0034.g125.t1	0	0	0	0.7	0	0.49	0	0	Ras-related protein Rab-35
Gastroderm	Collagen	aten_s0165.g33.t1	215.98	753.05	160.7	51.09	203.36	223.89	1.2	0.74	cGMP-inhibited 3′,5′-cyclic phosphodiesterase A
Gland/secretory cells	Trypsin	aten_s0043.g48.t1	0	0	0	0	0	14.85	0	0	Acid phosphatase type 7
Gland/secretory cells	Disintegrin	aten_s0045.g7.t1	14.73	3.65	3.53	13.56	4.06	6.56	0.89	0	Papilin
Gland/secretory cells	Trypsin	aten_s0184.g1.t1	0.29	4.08	37.05	0.28	3.28	3.53	0	0	Transmembrane protease serine 9
Gland/secretory cells	Trypsin	aten_s0058.g97.t1	0	1.2	0	0	0	0	0	0	RalA-binding protein 1
Gland/secretory cells	Trypsin	aten_s0061.g39.t1	0	0.87	0	23.97	0	0	2.76	0	Chymotrypsinogen B
Gland/secretory cells	Mucin	aten_s0085.g99.t1	15.8	1.2	13.2	22.16	5.69	17.37	0.25	0	E3 ubiquitin-protein ligase UBR4
Progenitors/undifferentiated cells	Histone H1	aten_s0210.g6.t1	0	0.17	0	0	0	0	0	0	Leucine-rich PPR motif-containing protein, mitochondrial
Progenitors /undifferentiated cells	Myc	aten_s0092.g59.t1	0	0.79	7.41	1.47	0	2.04	0.63	0	Myc proto-oncogene protein
Epidermis	Zona pellucida	aten_s0008.g106.t1	0	0.24	0	0	1.75	0	0	0	Oncoprotein-induced transcript 3 protein
Epidermis	Plac8	aten_s0207.g28.t1	0	0	0	0	0	0	0	42.26	Glycine betaine transporter OpuD
Neurons	Synaptotagmin	aten_s0014.g109.t1	1.11	0	0	0	0	1.87	0	0	Dolichol phosphate-mannose biosynthesis regulatory protein
Neurons	Synaptotagmin	aten_s0118.g10.t1	0	0	0	0.4	0	0	0	0	Synaptotagmin-15
Larval neuron	Shaw ion channel	aten_s0017.g96.t1	0	0	0	0	0	6.54	0	0	Calmodulin
Larval apical organ	Frizzled	aten_s0077.g16.t1	0	0	0	2.45	0	1.14	0	0	Smoothened homolog
Dig filaments	Aquaporin	aten_s0023.g15.t1	0.51	0	0	0.5	0	0	0	0	Lens fiber major intrinsic protein

#### Gene Ontology Profiles

Gene Ontology was applied to infer the properties of the in vitro cells. Four hundred sixty-eight of 676 genes were categorized under “*cellular component*,” “*biological function*,” and “*molecular function*,” and properties of the lines were inferred from these profiles. All eight lines exhibited similar GO patterns. That is, genes with *molecular function* were most abundant, genes with *biological function* next, and genes with *cellular component* were least abundant (Supplementary Fig. 2). Partly due to the single-cell-level analysis, the GO profile analysis did not always yield useful information to characterize the properties of each cell line.

#### Characterizing Properties of Cell Lines with Marker Genes

In *Nematostella vectensis*, another cnidarian, Sebe-Pedros et al. (2017) characterized nine cell types of larvae using 28 marker genes, including *collagen* and *ferritin* for markers of gastroderm; *plac8* and *zona pellucida* for the epidermis;* synaptotagmin*, *elav*, *rpamide*,* synapsin*, and *eag ion channel* for neurons*; Shaw ion channel* for larval neurons; *FGF1a* and *Frizzled* for larval apical organ; *nanos1*, *myc*, *neuroD*,* histone H1*,* CENP*,* soxB2a* and *septin* for progenitors/undifferentiated cells; *aquaporin *for Dig filaments;* carboxypeptidase A*, *trypsin*,* disintegrin*, *mucin*, and *chitin deacetylase* for glandular/secretory cells; and *spinalin*, *minicollagen*, and *nematogalectin* for cnidocytes. Employing these features as criteria, we examined whether the eight lines of in vitro cells express marker genes, and if so, which lines express which markers. We found specific and shared expression profiles of several marker genes in the cell lines (Table 2; Table S2).

First, IVB6 and IIC, brilliant brown cells, expressed marker genes for gastroderm and those for glandular/secretory cells (Table 2). However, they likely have different properties since the former expressed markers for neurons, while the latter produced markers for progenitors and epidermis. Expression of two neuron-related genes, *neuronal calcium sensor 1* and *neurogenic locus Notch*, was detected in IVB6 (Table S2).

Second, dark cell lines, IVB4, IVC4, and IVD1 expressed markers of gastroderm and glandular/secretory cells at high levels (Table 2). In addition, IVB4 cells also expressed *myc* (undifferentiation marker) (Table 2) and a photopigment, *melanopsin-A* abundantly (Table S2). On the other hand, IVC4 expressed neuronal markers (Table 1) and *melanotransferrin* (Table S2), whereas IVD1 expressed an epidermis marker (Table 1). Therefore, although all three lines have properties of gastroderm and glandular/secretory cells, each line has different properties as well.

Third, a gene for Shaw ion channel (larval neuron marker) was expressed only in IVC6 (cell-sheet) (Table 2). IVC6 cells also expressed *synaptotagmin* (neuron marker), *frizzled* (larval apical organ marker), and *Notch* (Table S2), suggesting that this cell line has neuronal properties. On the other hand, IVC6 expressed other genes, including those for collagen (gastroderm marker), disintegrin and trypsin (secretory cell markers), and also myc (undifferentiated cell marker). These results indicate that IVC6 comprises various cell types, but among the eight lines, these are the only cells with neuronal markers.

Fourth, *Plac8*, a novel placenta-specific gene (GenBank Accession, DC643839) that is used as an epidermis marker in *Nematostella*, is expressed only in IIIB6 (Table 2). IIIB6 is a gastrula-like cell cluster, in which an epidermis-like outer layer covers the cluster. The expression of *plac8* in the line indicates that the outer-layer cells of IIIB6 have properties of epidermis. IIIB6 also expresses a very low level of *collagen* genes, suggesting properties of gastroderm (Table 2). On the other hand, marker genes expressed in IIID5 were different from those of IIIB6. IIID5 expressed a marker (*collagen*) of gastroderm, markers (*trypsin, disintegrin* and *mucin*) of glandular/secretory cells, and *myc* of undifferentiated cells (Table 2). Therefore, IIID5 is distinct from IIIB6, and has the potential to form several cell types. All cell properties mentioned above were stably maintained throughout successive cell cultures.

Although further characterization is required for each of the eight lines, gene expression profiles at the single-cell level indicate that each has its own properties.

## Discussion

Here, we attempted to establish sustainable in vitro cell lines from planula larvae of the coral, *Acropora tenuis*, and succeeded in obtaining and maintaining several lines for future studies of coral biology.

### Improvement of Methods

#### TE Medium with Collagenase, a New Powerful Cell Dissociation Medium for *A. tenuis* Cells

 In Cnidaria, collagenase has been used for successful isolation of striated muscle tissues from jellyfish mesoglea (Schmid and Alder 1984). To the best of our knowledge, however, there are no reports indicating that collagenase is effective for dissociation to single cells. This study also showed that collagenase alone was inefficient for cell dissociation of planula larvae from this coral. We have neither cytochemical nor biochemical evidence for collagen in planula larvae, but it is possible that the larval mesoglea contains collagen, because culture cells dissociated from larvae express *collagen*.

In vertebrate cell culture, a culture medium containing trypsin and EDTA (TE) is the conventional tool for dissociation of cells from the substratum. TE is thought to digest calcium-dependent cell adhesion molecules such as L-CAM (Edelman 1986) and E-cadherin (Takeichi 1988). In this study, TE by itself was insufficient to dissociate planula cells, because much debris remained even after a 4-h treatment. In contrast, by adding collagenase to TE medium (TEC), complete planula cell dissociation became possible. We assume that in planula larvae, TEC acts both on calcium-dependent cell adhesion molecules and extracellular matrix.

#### Cell Culture Medium

Attempts at cell culture of the budding tunicate, *Polyandrocarpa misakiensis*, have shown that when seawater is used as part of the culture medium, both the osmotic pressure and pH should be lowered (Kawamura and Fujiwara 1995). A clonal cell line of marine invertebrates was successfully established by mixing modified seawater with DMEM containing 15% fetal bovine serum at a ratio of 5:1 (Kawamura and Fujiwara 1995). In the present study, the same seawater medium and DMEM (basal medium) were applied to an in vitro culture of *Acropora* planula cells. We only changed the ratio of media in the mixture to 9:1. In this culture medium, planula cells were maintained for 1–2 weeks and cell growth was observed temporarily. Simultaneously, it turned out that this cell culture medium is insufficient to establish serial cell lines in culture.

Recently, Conkling et al. (2019) reported that an amino-acid-optimized nutrient medium stimulates rapid in vitro cell division in several sponge species. The culture medium for *Acropora tenuis* cells shown in this study may contain a minimum requirement of nutrients. In the case of corals, *Acropora* species lack a gene for the enzyme, cystathionine ß-synthase, which is essential for cysteine biosynthesis (Shinzato et al. 2011, 2020). Therefore, it is possible to improve coral cell growth by optimizing amino acids or other components in the culture medium, which will be the objective of future studies.

#### Effectiveness of Modular Protease

Tunicate retinoic acid-inducible modular protease (TRAMP) has been found with the aid of differential display of *Polyandrocarpa* cells treated with retinoic acid (Ohashi et al. 1999). TRAMP exhibits growth-promoting activity in *Polyandrocarpa* cell lines in serum-free cell culture medium (Ohashi et al. 1999). In the fruit fly, *Drosophila melanogaster*, CL8, an imaginal disc cell line, has been established (Currie et al. 1988). CL8 requires insulin and fly extracts as additives to maintain cell growth, and additives are substituted at least in part by the cell culture-conditioned medium, or by enterokinase, another kind of modular protease (Kawamura et al. 1999). We administered enterokinase to dissociated planula cells in the basal medium and found it less effective in supporting in vitro growth of planula cells (unpublished result).

In contrast to enterokinase, plasmin, which is a modular protease that degrades fibrin in the blood, has various effects on planula cells in culture (Schuliga et al. 2018 for a recent review). Plasmin promotes cell proliferation by degrading growth-inhibitory polypeptides and releasing latent growth factors from the extracellular matrix. First, in planula cell culture, plasmin maintains brilliant, brown-colored cells in culture and induces cell growth (IVB6 and IIC5 lines). From a morphological viewpoint, brown cells are similar to in vivo endoderm precursor cells. The expression profile of molecular markers in brilliant brown cells indicates that this cell type has gastroderm and glandular or secretory cell properties in the adult polyp transcriptome, suggesting an affinity for in vivo endoderm, although further studies are required to determine the source of brown cells with more specific molecular probes. Endoderm precursor cells have been lost from 57-day-old and 61-day-old planula larvae, and instead, the endodermal cell layer (presumptive gastrodermis) underlies the ectoderm. Interestingly, in vitro cells dissociated from these larvae scarcely contain brown cells.

Second, plasmin stimulates differentiation of flattened amorphous cells (IVB4, IVC4 and IVD1 lines), which have never been observed in plasmin-free basal growth medium. Amorphous cells likely originated from brown cells, because intermediate types of cells are often observable in cell culture dishes. Expression profiles of molecular markers in amorphous cells indicates that they have properties of various cell types, including gastroderm, glandular or secretory cells, and progenitor cells. Third, plasmin induces in vitro morphogenesis that resembles embryonic blastulae (IIID5 line) and gastrulae (IIIB6 line), and cell sheets (IVC6) as well. Before morphogenesis, brown cells extend pseudopods and reaggregate. The epithelial transformation of free cells seemingly resembles in vivo endoderm formation, although no information is available as to the mechanism of endodermal cell adhesion. As mentioned, enterokinase is not effective in maintaining planula culture cells and promoting their growth in vitro, suggesting that the proteolytic activity of the protein is insufficient for planula cell culture. Therefore, a modular protease, plasmin, is essential for establishment of coral cell lines in vitro.

### Confirmation of Coral Origins of Cell Lines

To ensure that the in vitro cell lines we established were derived from *A. tenuis* and not from unknown contamination, we used two tools, immunocytochemistry with a coral-specific antibody and a transcriptomic approach using RNA-seq. In this study, we made an antibody to *A. tenuis* Snail (At-Snail) and confirmed that 24-day-old larvae expressed At-Snail protein in both ectodermal and endodermal cells. Signals were found in nuclei. Snail signals were also maintained in cultured cells (Fig. 7d, e), but expression was distributed throughout the cytoplasm for unknown reasons. In the related species, *A. millepora*, *Snail* gene expression was examined during embryonic development (Hayward et al. 2004). In situ hybridization showed that *Am-Snail* gene expression begins in the presumptive endodermal epithelium at the pre-gastrulation stage and persists in internal cells of the embryos (Hayward et al. 2004). Although results cannot be easily compared between the two *Acropora* species, *A. tenuis Snail* expression may commence in embryonic endoderm and then extend toward larval ectoderm. Using RNA-seq analysis, we also confirmed that most highly expressed genes in these lines were *A. tenuis* genes. Therefore, we may have established sustainable in vitro cell lines of corals.

### Properties of Cell Lines for Future Studies

Transcriptomic analysis of cell lines by RNA-seq was carried out at the level of single cells or single clusters of cells (e.g., IIIB6). Results obtained therefore not only clearly demonstrated the coral origin of the cells, but also suggested properties of cells, in contrast to broad, but non-specific information, such as GO annotation. As mentioned above, brilliant brown cells likely have properties of gastroderm and glandular or secretory cells, suggesting an affinity for in vivo endoderm. Amorphous cells have properties of various cell types, including gastroderm, glandular or secretory cells, and their progenitor cells. Cell sheets have properties of nervous system. Gastrula-like clusters show differentiation of epidermis-like cells. Therefore, an interesting research subject using these cell lines is to follow more precisely the differentiation processes of planula cells in vitro. In addition, other studies are now ongoing with different cellular and molecular methods to reveal the potential of coral planula larval cells.

## Supplementary Information

Below is the link to the electronic supplementary material.

Supplementary file1 Supplementary Figure S1. Western blotting analysis of AtSnail (A) and anti-AtFat1 (B) antibodies. Lane 1, Total proteins isolated from planula larvae. Lane 2, Proteins isolated from cultured cells. Asterisk in (A) indicates a band corresponding to expected proteins. Asterisk in (B) indicates a band having lower molecular weight than expected proteins. M, Molecular weight markers. (JPG 173 KB)

Supplementary file2 Supplementary Figure S2. GO counts for genes of each cell line. RNA-seq analysis identified 676 genes with annotated functions. Of these, 36 genes were expressed in all eight lines while 640 genes were expressed preferentially and/or exclusively in a certain line. Specifically, 54 genes are specific to IVB6, 71 to IIC5, 61 to IVB4, 120 to IVC4, 53 to IVD1, 146 genes to IVC6, 38 to IIID5, and 31 to IIIB6. Those genes are listed in Supplementary Table 1. Most cell lines show a similar ratio of GO categories. (PDF 160 KB)

Supplementary file3 (XLSX 55 KB)
