# Supplementary figures and images for: Establishing Sustainable Cell Lines of a Coral, Acropora tenuis
Source: Mar Biotechnol (NY). 2021 Apr 26;23(3):373–88. doi: 10.1007/s10126-021-10031-w (PMC8270879; doi:10.1007/s10126-021-10031-w)

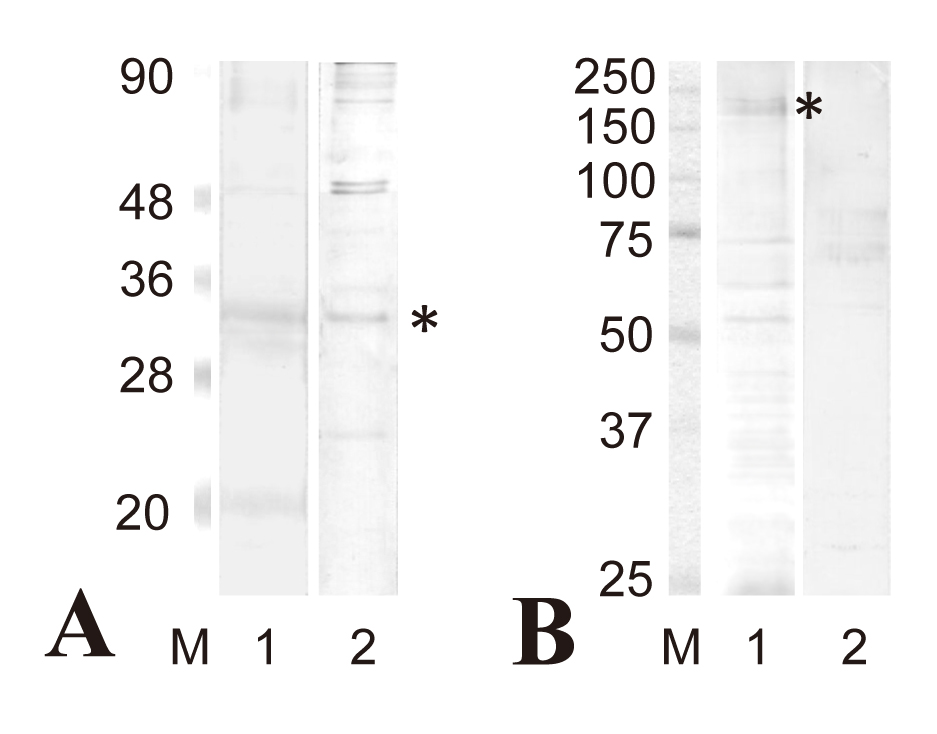

Supplement: Supplementary file 1 — Supplementary file1 Supplementary Figure S1. Western blotting analysis of AtSnail (A) and anti-AtFat1 (B) antibodies. Lane 1, Total proteins isolated from planula larvae. Lane 2, Proteins isolated from cultured cells. Asterisk in (A) indicates a band corresponding to expected proteins. Asterisk in (B) indicates a band having lower molecular weight than expected proteins. M, Molecular weight markers. (JPG 173 KB) [file 10126_2021_10031_MOESM1_ESM.jpg]
