# Supplementary material for: Establishing Sustainable Cell Lines of a Coral, Acropora tenuis
Source: Mar Biotechnol (NY). 2021 Apr 26;23(3):373–88. doi: 10.1007/s10126-021-10031-w (PMC8270879; doi:10.1007/s10126-021-10031-w)
Supplement: Supplementary file 2 — Supplementary file2 Supplementary Figure S2. GO counts for genes of each cell line. RNA-seq analysis identified 676 genes with annotated functions. Of these, 36 genes were expressed in all eight lines while 640 genes were expressed preferentially and/or exclusively in a certain line. Specifically, 54 genes are specific to IVB6, 71 to IIC5, 61 to IVB4, 120 to IVC4, 53 to IVD1, 146 genes to IVC6, 38 to IIID5, and 31 to IIIB6. Those genes are listed in Supplementary Table 1. Most cell lines show a similar ratio of GO categories. (PDF 160 KB) [file 10126_2021_10031_MOESM2_ESM.pdf]

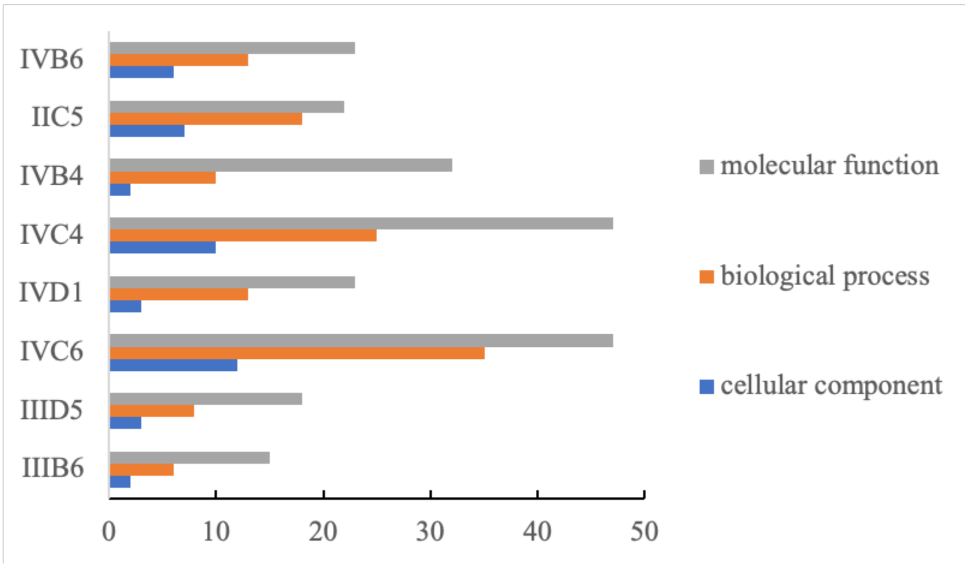

Supplementary Figure 2. GO counts for genes of each cell line. RNA-seq analysis identified 676 genes with annotated function. Of them, 36 genes were expressed commonly in all the eight lines while 640 genes were expressed preferentially and/or specifically in a certain line. Namely, 54 gene are specific to IVB6, 71 to IIC5, 61 to IVB4, 120 to IVC4, 53 to IVD1, 146 genes to IVC6, 38 to IIID5, and 31 to IIIB6. Those genes are listed up in Supplementary Table 1. Most cell lines show a similar ratio of GO categories.
